# Embedding Monodisperse LaO*_x_* Into Pt Nanoclusters for Ultra‐Stable and Efficient Hydrogen Isotope Oxidation

**DOI:** 10.1002/advs.202504224

**Published:** 2025-05-29

**Authors:** Guilin Wei, Jiangfeng Song, Yan Shi, Linsen Zhou, Xianglin Wang, Junhong Luo, Ning Liu, Feize Li, Xingwen Feng

**Affiliations:** ^1^ Institute of Materials China Academy of Engineering Physics Jiangyou Sichuan 621908 P. R. China; ^2^ Key Laboratory of Radiation Physics and Technology of the Ministry of Education Institute of Nuclear Science and Technology Sichuan University Chengdu 610064 P. R. China

**Keywords:** catalytic oxidation, hydrogen isotopes, platinum, silicalite‐1 zeolite, singl‐atom oxide

## Abstract

Catalytic oxidation plays a crucial role in the efficient treatment of hydrogen isotopes, with the key technical challenge being the development of high‐performance catalysts to enhance isotope removal efficiency, thereby reducing environmental pollution and ensuring public radiation safety. Herein, the strategic control of platinum nanoclusters confined within silicalite‐1 zeolites is reported to enhance the high removal efficiency for hydrogen isotopes under low‐temperature conditions for the first time. Incorporating a single lanthanum oxide (LaO_x_) into Pt nanoclusters adapts the local charge of adjacent Pt atoms, significantly altering their electronic structure. The existence of individual LaO_x_ species is confirmed by X‐ray absorption spectroscopy and aberration‐corrected electron microscopy, and the performance enhancement mechanism is further probed by theoretical calculations. This embedded single LaO_x_ both facilitates local charge adjustment and supplies abundant active oxygen species, leading to performance enhancement by reducing the energy barrier of rate‐determining step (O^*^ + H → OH^*^), in accordance with the Mars‐van Krevelen mechanism. Consequently, during a surprisingly long‐term restart performance test (≈267 d), the PtLaOx@S‐1 maintained a high conversion rate over 99% at 50 °C and a space velocity of 48,000 mL·g^−1^·h^−1^. This study highlights the potential of individual LaO_x_ sites for enhancing hydrogen isotope oxidation.

## Graphical abstract

The introduction of isolated LaO_x_ species into Pt nanoclusters confined within the silicalite‐1 zeolite significantly enhanced the oxidative removal of hydrogen isotopes. Under reaction conditions of 50 °C and GHSV of 72,000 mL·g^−1^·h^−1^, the catalyst achieved an excellent H_2_ conversion efficiency of over 99%. Theoretical calculations reveal that the synergistic effect of LaO_x_ substantially lowers the reaction energy barrier.

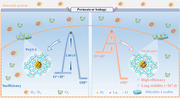

## 1 Introduction

Tritium (*T*_1/2_ = 12.3 years) is radioactive and its release could pose significant risks to human health and the environment.^[^ ^1^, ^2^, ^3^ ^]^ The oxidation reaction of hydrogen isotopes (hydrogen, deuterium, and tritium) is a critical process for ensuring tritium safety.^[^ ^4^ ^]^ Traditional platinum‐based catalysts have garnered extensive attention due to their high efficiency in hydrogen isotope oxidation reactions. However, these catalysts are prone to significant cluster migration at higher hireaction temperatures (>150 °C), leading to a noticeable decline in catalytic performance.^[^ ^5^, ^6^ ^]^ Additionally, at lower temperatures, the catalysts exhibit poor ability to activate O_2_ due to the need to overcome higher activation energy barriers, resulting in lower catalytic efficiency.^[^ ^7^ ^]^ With the increasing stringency of tritium safety regulations and the need for rapid response under accident conditions, there is an urgent demand for catalysts capable of efficiently oxidizing hydrogen isotopes at low temperatures.^[^ ^8^, ^9^, ^10^ ^]^

The confinement effect of zeolites has been proven effective in anchoring active sites within the framework structure, thereby suppressing their migration at high temperatures and mitigating Ostwald ripening.^[^ ^11^ ^]^ Platinum metal nanocluster catalysts confined within zeolites, synthesized through a ligand‐protected one‐pot hydrothermal method, have been successfully applied to the catalytic oxidation of hydrogen isotopes.^[^ ^12^ ^]^ Moreover, the catalytic performance of platinum‐based zeolite catalysts can be further enhanced through a series of microstructural tuning strategies, including metal doping,^[^ ^13^, ^14^, ^15^, ^16^ ^]^ thermal treatments under different atmospheres,^[^ ^17^, ^18^, ^19^, ^20^ ^]^ and support modification.^[^ ^21^, ^22^, ^23^ ^]^ Among these, the metal doping strategy is particularly effective, as it introduces a second non‐noble metal to fine‐tune the microenvironment of active sites,^[^ ^24^, ^25^, ^26^ ^]^ such as Pt‐Y,^[^ ^27^ ^]^ Pt‐Ce,^[^ ^28^ ^]^ and Pt‐Co.^[^ ^29^ ^]^ Therefore, the strategy of confining bimetallic sites within the micropores of zeolites can significantly enhance catalytic activity.^[^ ^30^, ^31^ ^]^ Lanthanide oxides, due to their unique electronic structures, have also been employed as modifiers for noble metals.^[^ ^32^, ^33^ ^]^ Lanthanum (La), as a representative lanthanide metal, has been extensively studied owing to its distinctive electronic structure and the abundance of surface oxygen species. From the perspective of alloys, La is a suitable element due to its electronic effects, geometric effects, redox properties, and synergistic interactions with Pt. These attributes make Pt─La active sites promising catalysts for various catalytic reactions. Feng et al. found that the interaction between Pt and La alters the electronic structure of the catalyst, significantly enhancing the catalytic performance in methanol oxidation, hydrogen evolution, and their coupling reactions.^[^ ^34^ ^]^ Lu et al. discovered that surface modification of La leads to an increase in the number of moderate basic sites and surface oxygen vacancies in the PtLa/CeO_2_ catalyst, resulting in a significant boost in hydrogen production during methanol aqueous reforming.^[^ ^35^ ^]^ Fan et al. studied the formation process of Pt_5_La nanoalloys and characterized their oxygen reduction reaction and methanol oxidation reaction catalytic performance in acidic solutions.^[^ ^36^ ^]^ Although extensive research has been conducted on Pt─La active sites, studies focusing on Pt─La sites confined within zeolites, particularly those where La atoms exist in a single‐dispersed state, remain limited. Furthermore, there is a lack of in‐depth investigation into their performance and mechanisms in hydrogen isotope oxidation applications. First, what is the performance of zeolite‐confined Pt─La sites in hydrogen isotope oxidation? Second, what is the role of La sites throughout the hydrogen isotope oxidation process? Thirdly, what is the dominant mechanism of hydrogen isotope oxidation over Pt─La based catalysts? These questions pertain to the exploration of active sites for hydrogen isotope oxidation and the establishment of structure‐activity relationships between the elements involved.

Herein, a universal ligand‐protected hydrothermal strategy was employed to successfully synthesize bimetallic cluster catalysts confined within hydrophobic silicalite‐1 (S‐1) zeolites. In this system, Pt nanoclusters were modified with monodisperse lanthanum oxide (LaO*_x_*) species in S‐1 (PtLaO*_x_* @S‐1) and applied for the first time to the catalytic oxidation of hydrogen isotopes. Multiple characterizations including aberration‐corrected electron microscopy and synchrotron radiation absorption spectroscopy reveal distinct structural features of single LaO*_x_* units embedded within the Pt nanoclusters (PtLaO*_x_*). The mechanistic role of PtLaO*_x_* active sites in the catalytic oxidation of hydrogen isotopes was probed through theoretical calculations. The study demonstrates that the active sites in PtLaO*_x_* @S‐1 exhibit a lower energy barrier (only 0.44 eV) for the rate‐determining step (RDS), significantly improving the reaction efficiency following the Mars‐van Krevelen mechanism.^[^ ^37^ ^]^ Under conditions of 50 °C and a high gas hourly space velocity (GHSV) of 72000 mL·g^−1^·h^−1^, the catalyst achieved a highly stable conversion rate of >99% for H_2_ (D_2_) and maintained high performance during a surprisingly long‐term restart test spanning 267 d. Furthermore, after exposure to 300 kGy of *β*‐radiation, the catalyst exhibited only a ≈1% decline in efficiency, showing exceptional radiation resistance. Combined with the performance results of Ce‐ and Pr‐doped Pt‐based S‐1 zeolite catalysts, the findings indicate that lanthanide oxide doping can significantly enhance the stability and activity of Pt‐based zeolite catalysts.

## 2 Results and Discussion

**Figure 1:**
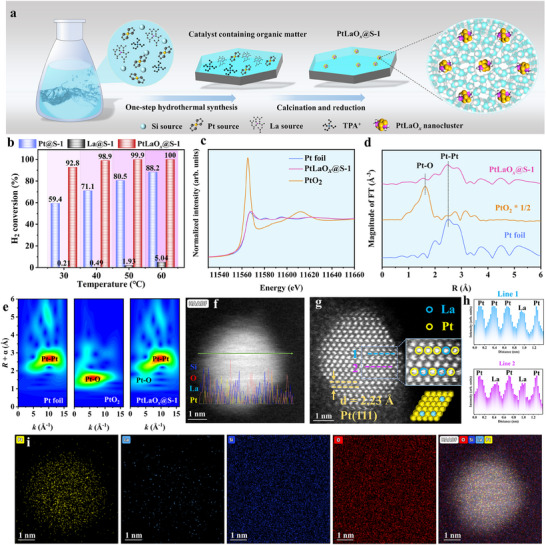
Preparation, characterization, and performance testing of PtLaO*_x_* @S‐1. a) Schematic illustration on the synthesis of PtLaO*_x_* @S‐1. b) The catalytic oxidation conversion rate of H_2_ by Pt@S‐1, La@S‐1, and PtLaO*_x_* @S‐1. Experimental conditions: 0.05 g catalyst, different temperatures (30, 40, 50, and 60 °C), GHSV = 48000 mL·g^−1^·h^−1^, 2000 ppm H_2_. c) Pt L_3_‐edge XANES spectra of Pt foil, PtO_2_, and PtLaO*_x_* @S‐1. d) EXAFS spectra of Pt foil, PtO_2_, and PtLaO*_x_* @S‐1. e) WT‐EXAFS of Pt foil, PtO_2_, and PtLaO*_x_* @S‐1. f) HAADF‐STEM image (inset: line scan of elemental profiles) of PtLaO*_x_* @S‐1. g) Atomic‐resolution HAADF‐STEM image of PtLaO*_x_* @S‐1 (inset: the atomic structure model). h) Intensity profiles along lines 1 and 2 indicated in h). i) STEM‐EDX mapping images of PtLaO*_x_* @S‐1 showing distributions of Pt (yellow), La (sky blue), Si (dark blue), O (red), and merged elements.

Three types of catalysts (Pt@S‐1, La@S‐1, and PtLaO*_x_* @S‐1) were synthesized in one step using a ligand‐protected hydrothermal method (**Figure** **1**a). Based on the TG‐DSC results, the calcination temperature in air was determined to be 550 °C (Figure S1, Supporting Information). The Pt content in obtained Pt@S‐1 was 0.56 wt.% while the La content in La@S‐1 was 0.11 wt.%, after subsequent reduction treatment. The Pt and La contents in PtLaO*_x_* @S‐1 were 0.55 and 0.13 wt.%, respectively (Table S1, Supporting Information). The X‐ray diffraction (XRD) patterns and Fourier transform infrared (FT‐IR) spectra results indicate that the obtained catalyst exhibits the characteristic structure of an MFI‐type zeolite (Figures S2,S3, Supporting Information). Using the hydrogen isotope gas catalytic oxidation apparatus (Figure S4, Supporting Information), the catalytic oxidation conversion rates of H_2_ over Pt@S‐1, La@S‐1, and PtLaO*_x_* @S‐1 were evaluated at a fixed gas hourly space velocity (GHSV) of 48000 mL·g^−1^·h^−1^ and different temperatures (30, 40, 50, and 60 °C). As presented in Figure 1b, Pt@S‐1 achieved H_2_ conversion rates of 59.4%, 71.1%, 80.5%, and 88.2% at 30, 40, 50, and 60 °C, respectively. In contrast, the La@S‐1 exhibited negligible H_2_ conversion across all tested temperatures, indicating that the catalyst lacks active sites for the reaction when only La element is present. Interestingly, when La was doped into Pt@S‐1, the resulting PtLaO*_x_* @S‐1 achieved H_2_ conversion rates of 92.8%, 98.9%, 99.9%, and 100% at 30, 40, 50, and 60 °C, respectively. This result demonstrates that the incorporation of La significantly enhances the conversion efficiency of Pt‐based S‐1 zeolite catalyst. This doping effect becomes more pronounced under low‐temperature reaction conditions. For instance, at 30 °C, the H_2_ conversion rate of the PtLaO*_x_* @S‐1 exceeds that of the Pt@S‐1 by 33.4%. The microstructural characteristics of Pt@S‐1 have been investigated by transmission electron microscopy (TEM), indicating that it has the typical hexagonal morphology of MFI zeolite (Figure S5, Supporting Information).^[^ ^38^, ^39^ ^]^ High‐resolution transmission electron microscopy (HRTEM) reveals the presence of Pt metal clusters in Pt@S‐1. The lattice spacing of the clusters is ≈2.27 Å, corresponding to the Pt(111) crystal plane.^[^ ^40^, ^41^ ^]^ Energy‐dispersive spectroscopy (EDS) provides the elemental distribution of Pt, O, and Si. High‐angle annular dark‐field scanning transmission electron microscopy (HAADF‐STEM) suggests that the average particle size of the Pt clusters was 3.4 nm. The HRTEM results of the PtLaO*_x_* @S‐1 catalyst reveal the presence of metal clusters, with an average cluster size of 3.1 nm (Figure S6, Supporting Information). A comparison of particle size results indicates that the incorporation of La has no obvious effect on the size of the formed clusters. Additionally, the Pt(111) crystal planes can also be observed in PtLaO*_x_* @S‐1, suggesting a close correlation between the doped La and this crystal facet. From the microscopic morphology analysis of La@S‐1 (Figure S7, Supporting Information), no obvious metal clusters were observed. Combined with the La content (0.11 wt.%) determined by ICP‐OES, it can be inferred that La atoms are likely highly dispersed as single atoms within the zeolite framework rather than forming observable nanoclusters. This phenomenon is consistent with the results from previously reported work.^[^ ^22^, ^42^ ^]^ A similar situation may also exist in the PtLaO*_x_* @S‐1, where a portion of the La atoms could present inside the zeolite framework. However, based on the catalytic performance of La@S‐1, these La atoms show no apparent activity in catalyzing of H_2_ oxidation. Overall, a comparison of the catalytic performance of Pt@S‐1, La@S‐1, and PtLaO*_x_* @S‐1 preliminarily indicates that the significant enhancement in the H_2_ conversion rate of the designed PtLaO*_x_* @S‐1 in tightly to incorporating La into the Pt nanoclusters.

X‐ray Absorption Near Edge Structure (XANES) and Extended X‐ray Absorption Fine Structure (EXAFS) measurements were conducted to evaluate the coordination environment and structural characteristics of Pt in PtLaO*_x_* @S‐1. Figure 1c shows the Pt L_3_‐edge XANES spectra of PtLaO*_x_* @S‐1 and the reference materials (Pt foil and PtO_2_). The white line intensity of PtLaO*_x_* @S‐1 is located between that of Pt foil (Pt^0^) and PtO_2_ (Pt^4+^), indicating that the average oxidation state of Pt is partially positive. This is attributed to the transfer of electrons from Pt to the O atoms in the zeolite structure.^[^ ^43^, ^44^ ^]^ Additionally, Fourier transform (FT) of the Pt L_3_‐edge EXAFS spectra was performed to elucidate the bonding information. A dominant peak characteristic of Pt foil and a secondary, more intense peak resembling that of PtO_2_ are observed in corresponding spectrum of PtLaO*_x_* @S‐1 (Figure 1d). These peaks can be attributed to the contributions from Pt─Pt and Pt─O interactions, respectively. The FT‐EXAFS curve of the Pt L_3_‐edge was fitted in R‐space (Figure S8 and Table S2 Supporting Information). Compared to Pt foil (12), the average Pt─Pt coordination number (CN) in PtLaO*_x_* @S‐1 (4.7) is lower, primarily due to the confinement of Pt in the form of nanoclusters within the zeolite structure. Additionally, to obtain more direct bonding information, we performed wavelet transform (WT) analysis (Figure 1e). The results show that PtLaO*_x_* @S‐1 exhibits prominent peaks corresponding to both Pt foil and PtO_2_, consistent with the EXAFS results. Unfortunately, due to the low La content in PtLaO*_x_* @S‐1, the XANES analysis did not reveal a considerable La signal.

To determine the form in which La exists in the catalyst, the metallic components of PtLaO*_x_* @S‐1 were comprehensively analyzed through aberration‐corrected high‐angle annular dark‐field scanning transmission electron microscopy (AC‐HAADF‐STEM). The low‐magnification HAADF‐STEM image reveals that the cluster particle size is ≈3.2 nm (Figure S9, Supporting Information), which is consistent with the result from HRTEM (Figure S6, Supporting Information). The line scan profile in Figure 1f and the elemental mapping results (Figure S10, Supporting Information) confirm that the embedded nanoclusters are composed of a Pt─La bimetallic alloy. The AC‐HAADF‐STEM image of PtLaO*_x_* @S‐1 further reveals that La atoms are embedded among Pt atoms in an atomically dispersed form (Figure 1g). The intensity distribution shows distinct variations in Z‐axis contrast between Pt and La atoms along Line 1 in Figure 1h, highlighting the spatial arrangement of Pt and La atoms within PtLaO*_x_* @S‐1. Moreover, the AC‐HAADF‐STEM image also suggests a lattice spacing of 2.23 Å, corresponding to the characteristic Pt(111) plane. It is noteworthy that the La content observed in the nanoclusters via AC‐HAADF‐STEM is relatively low and Pt is predominant component, this appears to be inconsistent with the Pt/La ratio determined by ICP‐OES analysis. However, according to above microscopic morphology analysis of La@S‐1, this discrepancy may be attributed to the partial incorporation of La inside the zeolite framework. The energy‐dispersive X‐ray (EDX) elemental mapping in Figure 1i demonstrates the evenly spatial distribution of Pt, La, Si, and O elements. Based on all these results, it can be confirmed that part of La is inserted as single atoms within the Pt nanoclusters on the (111) crystal plane, as illustrated in the inset in Figure 1g.

**Figure 2:**
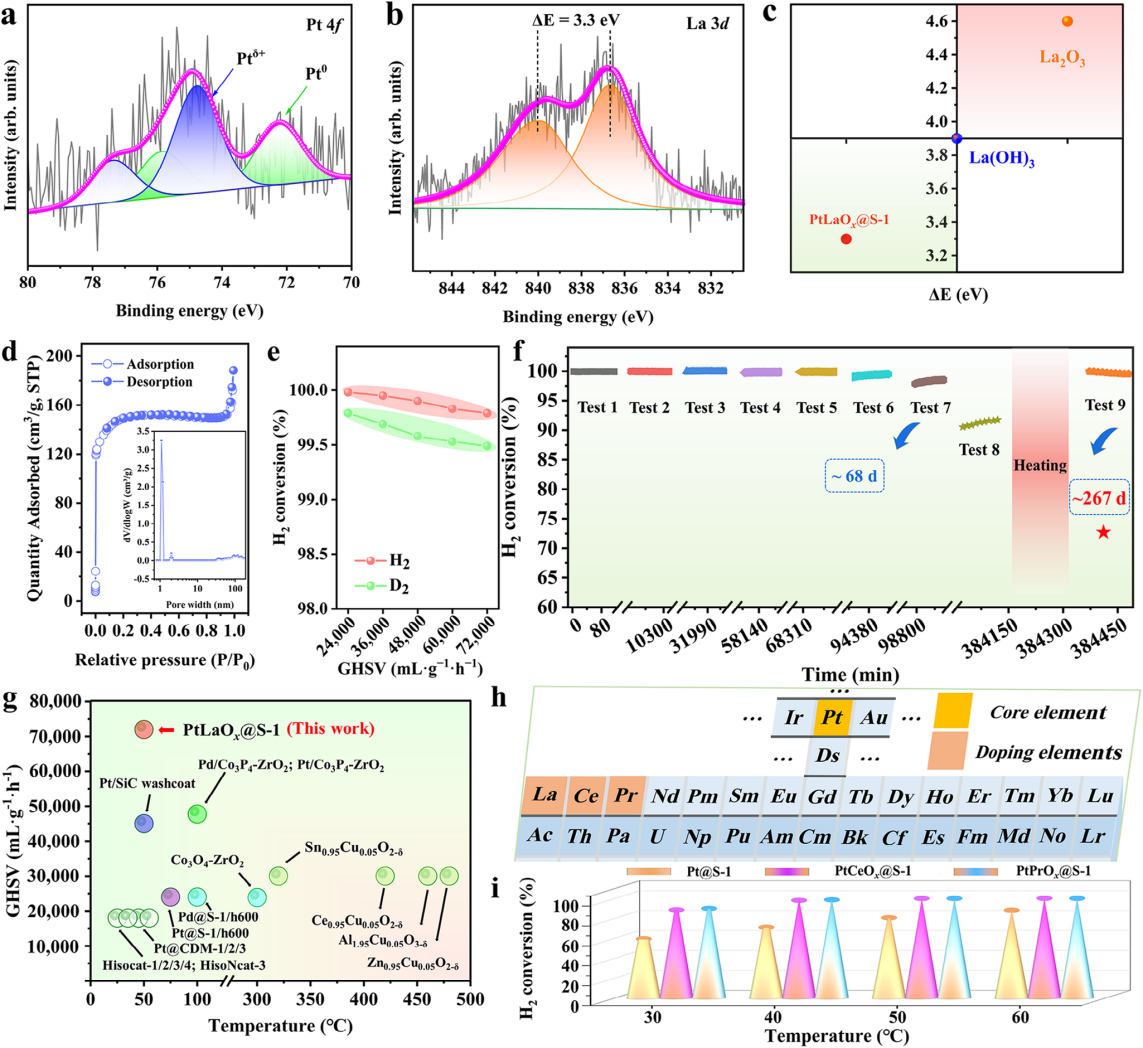
Characterization and performance testing of PtLaO*_x_* @S‐1. a) Pt 4*f* XPS spectra of PtLaO*_x_* @S‐1. b) La 3*d* XPS spectra of PtLaO*_x_* @S‐1. c) Δ*E* comparison for La between PtLaO*_x_* @S‐1 and reported La(OH)_3_ and La_2_O_3_.^[^ ^48^ ^]^ d) BET and pore size distribution of PtLaO*_x_* @S‐1. e) The catalytic oxidation conversion rate of H_2_ and D_2_ (2000 ppm) by PtLaO*_x_* @S‐1. Experimental conditions: 0.05 g catalyst, 50 °C, different GHSV conditions (24000, 36000, 48000, 60000, 72000 mL·g^−1^·h^−1^). f) Restart performance test of PtLaO*_x_* @S‐1. Experimental conditions: 0.05 g catalyst, 50 °C, GHSV = 48000 mL·g^−1^·h^−1^. g) Comparison of the temperature and gas hourly space velocity (GHSV) parameters of the reported catalysts and the PtLaO*_x_* @S‐1 in achieving a conversion rate of ≥99%. h) Ce and Pr were selected as doping elements to prepare catalysts PtCeO*_x_* @S‐1 and PtPrO*_x_* @S‐1, and i) their H_2_ conversion results. Experimental conditions: 0.05 g catalyst, different temperatures (30, 40, 50, and 60 °C), GHSV = 48000 mL·g^−1^·h^−1^.

X‐ray photoelectron spectroscopy (XPS) results of PtLaO*_x_* @S‐1 have been characterized (Figure S11, Supporting Information). **Figure** **2**a shows the XPS result of the Pt 4*f* for PtLaO*_x_* @S‐1, revealing that the Pt species in the catalyst primarily include two states (Pt^δ+^ and Pt^0^). The presence of Pt^δ+^ can result from two factors. The first is electron transfer between Pt atoms in the Pt nanoclusters and oxygen atoms in the zeolite structure.^[^ ^43^, ^44^ ^]^ The second possible reason is electron transfer between Pt atoms and the doped La atoms. Pt^0^ corresponds to metallic Pt atoms. In the case of the La 3*d* region (Figure 2b), doublet peaks at 836.61 and 838.91 eV could be observed, which originates from the oxide state.^[^ ^45^ ^]^ Moreover, the spin‐orbit splitting energy of PtLaO*_x_* @S‐1 is 3.3 eV, compared to 3.9 eV for La(OH)_3_ and 4.6 eV for La_2_O_3_ (Figure 2c).^[^ ^46^, ^47^ ^]^ Notably, the reduced splitting energy of La indicates a decrease in its electron cloud density, suggesting the presence of electron transfer between La, Pt, and O atoms.^[^ ^48^ ^]^ Furthermore, PtLaO*_x_* @S‐1, which exhibits a favorable porous structure (BET surface area of 467.1 m^2^ g^−1^, Figure 2d), was evaluated for its catalytic oxidation performance of H_2_ and D_2_ under different GHSV conditions. As shown in Figure 2e, PtLaO*_x_* @S‐1 exhibited conversion rates of over 99% for H_2_ and D_2_ under GHSV conditions of 24000, 36000, 48000, 60000, and 72000 mL·g^−1^·h^−1^ at 50 °C. The long‐term restart performance of PtLaO*_x_* @S‐1 over a cumulative period of 267 d was evaluated (Figure 2f). During the first 68 d, seven restart performance tests were conducted, with the conversion efficiency slightly decreasing from 99.9% to 98.5%. After being exposed to air for ≈267 d, the performance under the same experimental conditions declined to 91.8%. Despite this reduction, the catalytic efficiency could be restored to over 99% following a simple inert gas heating treatment at 400 °C. XRD, TEM, and particle size analysis after prolonged use confirmes that the catalyst retained good structural stability after multiple cycles of use (Figures S12,S13, Supporting Information). Figure 2g compares the threshold temperature and GHSV (refer to Table S3, Supporting Information for details) at which different catalysts achieve an H_2_ conversion rate ≈99%. It indicates that PtLaO*_x_* @S‐1 exhibits certain advantages under high GHSV and relatively low‐temperature conditions. To confirm that doping with lanthanide elements can serve as a general strategy for promoting the catalytic oxidation of hydrogen isotopes by platinum‐based zeolites, two other catalysts doped with Ce and Pr, PtCeO*_x_* @S‐1 and PtPrO*_x_* @S‐1, respectively, were synthesized (Figure 2h; Figures S14–S17, Supporting Information). The H_2_ conversion rates of both catalysts were evaluated under different temperatures and a GHSV of 48000 mL·g^−1^·h^−1^. As shown in Figure 2i, the prepared PtCeO*_x_* @S‐1 exhibited considerable hydrogen conversion efficiencies of 88.5%, 98%, 99.9%, and 100% at 30, 40, 50, and 60 °C, respectively. Similarly, the obtained PtPrO*_x_* @S‐1 achieved high hydrogen conversion efficiencies of 89.9%, 98.6%, 99.9%, and 100% at respective temperatures. In comparison to the lower performance of Pt@S‐1 under identical experimental conditions, the enhanced catalytic performance observed after doping with Ce and Pr suggests that lanthanide metal doping has good universality to improve the hydrogen isotope removal efficiency of platinum‐based zeolite catalysts.

**Figure 3:**
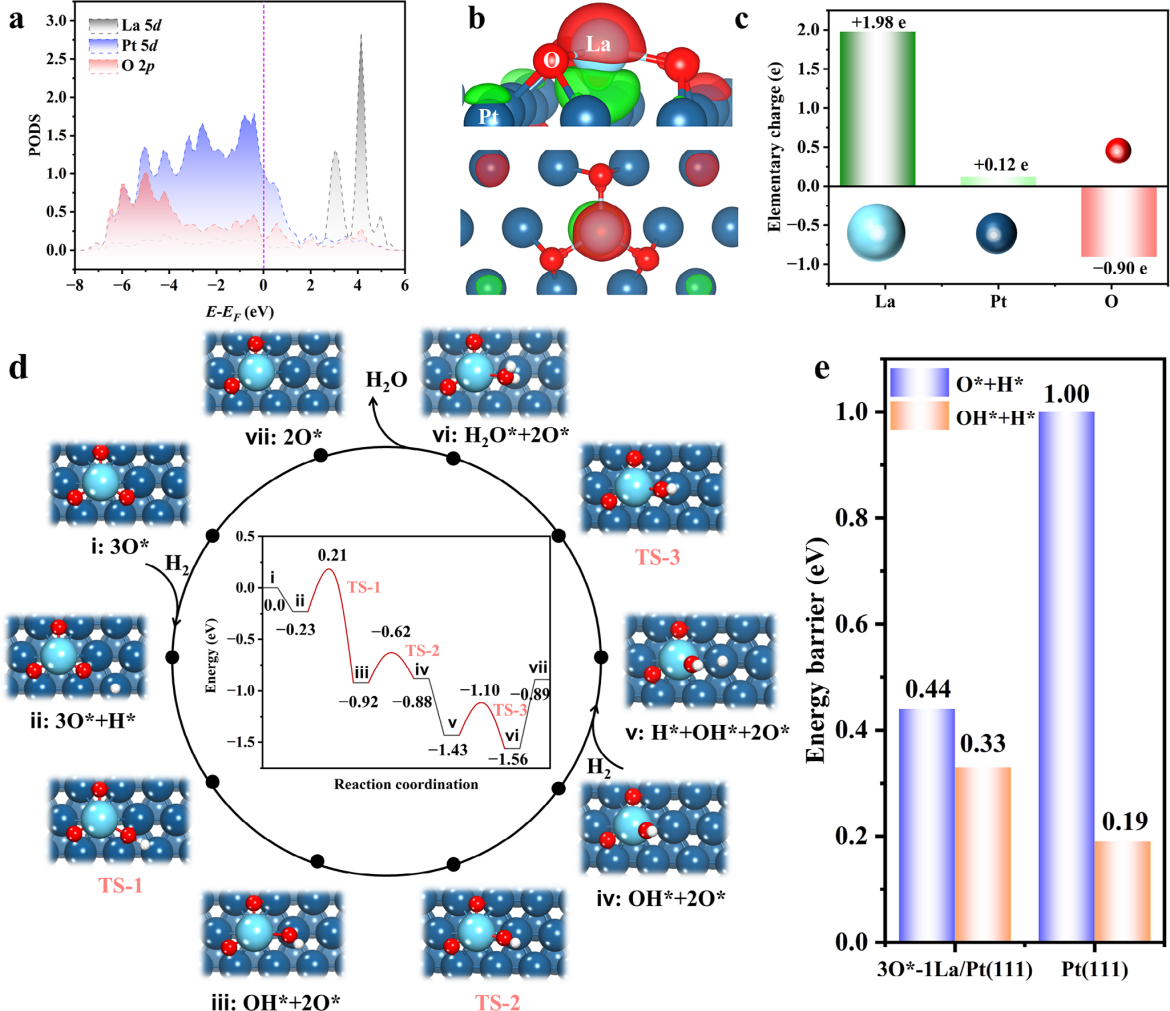
Analysis of catalytic mechanism. a) The partial density of states (PDOS) of 3O^*^‐1La/Pt(111) structure. b) The charge density differences of 3O^*^‐1La/Pt(111) structure. c) Net charge values on individual La, Pt, and O atoms. d) The reaction energy profiles and reaction pathway of oxidation H_2_ on 3O^*^‐1La/Pt(111). e) Comparison of reaction energy barriers between 3O^*^‐1La/Pt(111) and Pt(111).

Theoretical calculations were conducted to explore the mechanism of hydrogen isotope catalytic oxidation reaction mechanisms on the PtLaO*_x_* @S‐1 catalyst. Based on the combined results of aberration‐corrected electron microscopy, synchrotron radiation absorption spectroscopy, and XPS, 1La/Pt(111) slab model for the single‐atom La embedded Pt(111) surface was constructed (Figure S18, Supporting Information). The partial density of states (PDOS) of Pt 5*d* and La 5*d* were calculated (Figure S19, Supporting Information). The hybridization between the Pt 5*d* and La 5*d* states can be observed in a wide energy range from −7.0 to 6.0 eV, where the PDOS of Pt 5*d* is dominant in the valence bands and those of La 5*d* becomes dominant in the conduction bands. The charge density difference for 1La/Pt(111) surface was evaluated (Figure S20, Supporting Information). Due to the higher electronegativity of Pt (≈2.2) than La (≈1.0–1.2),^[^ ^49^ ^]^ there are obviously charge transferred from La atoms to the surrounding Pt atoms. The La atom possesses a positive net charge of +1.61 e, in contrast to those nearby Pt atoms with a negative net charge of −0.25 e. Subsequently, La atom can serve as the Lewis acid to regulate the adsorption behaviors of O and OH species.^[^ ^48^ ^]^ The adsorption energies (*E*_ads_) of the hydroxyl molecule (OH^*^) and oxygen atom (O^*^) at the top, fcc, and hcp sites were calculated using the formula *E*_ads_ = *E*_total_−*E*_surface_−*E*_gas_, where *E*_total_, *E*_surface_, and *E*_gas_ are the energies of the total system, pure surface, and gas species (*E*_OH_ or 1/2E_O2_), respectively (Figure S21, Supporting Information). The OH molecule exhibits a vertical adsorption configuration on the La‐top site and becomes a tilted adsorption configuration on the neighbor Pt‐top site, which have an adsorption energy of −3.44 and −3.33 eV, respectively, in comparison with that of −2.33 eV for the tilted top configuration on pure Pt(111) surface.^[^ ^50^ ^]^ Moreover, the adsorption energies of the oxygen atom at the La‐top, fcc, and hcp sites are 0.19, −1.78, and −1.58 eV, respectively. Such that the oxygen atom prefers to adsorb at the fcc site with one La atom and two Pt atoms, which have the adsorption energy (−1.78 eV) stronger than that of ≈−1.3 eV on the fcc site of pure Pt(111) surface.^[^ ^50^ ^]^ Besides, the XPS results indicate that La exists in an oxidized state, prompting consideration of the plausible positions of oxygen atoms around the La atoms. According to the theoretical and experimental results, the La atom exhibits high oxophilicity, and a 3O^*^‐1La/Pt(111) surface was modeled with the three fcc sites around La atom fully covered with O^*^ adatoms. The PDOS of Pt 5*d*, La 5*d*, and O 2*p* states are shown in **Figure** **3**a, where the O 2*p* states overlap with the surface‐d orbitals between −7.0 and 5.0 eV. 3O^*^‐1La/Pt(111) has an average adsorption energy of −1.77 eV, which is similar to that of one O^*^ on 1La/Pt(111) (Figure S21, Supporting Information). This indicates that there are few repulsive forces among three O^*^ adatoms, and highly oxophilic interaction for La atom. Moreover, Figure 3b presents the charge density difference for 3O^*^‐1La/Pt(111) surface. After oxygen adsorption, both La and Pt surface atoms lost electrons with a positive charge of +1.98 and +0.12 e, respectively, while O adatoms gain electron with a negative charge of −0.90 e (Figure 3c). These are a total charge transfer of 2.70 e from the surface to 3O^*^ adsorbates, leading to significantly charge redistribution on the surface, which is consistent with the reduction in electron density observed in the XPS analysis of La element. Therefore, the 3O^*^‐1La/Pt(111) oxidization surface was utilized to study the mechanism of catalytic H_2_ oxidation.

Figure 3d shows the reaction pathway for hydrogen oxidation on the 3O^*^‐1La/Pt(111) surface. The first step is the combination of one O^*^ surface atom with an adsorbed H^*^ atom (*E*_ads_ = −0.23 eV) at the nearby Pt‐top site to form the titled OH^*^, which proceeds with an energy barrier (TS‐1) of 0.44 eV and a reaction energy of −0.69 eV. The second step is the migration of the tilted OH^*^ on Pt‐top site to the vertical one on La‐top site, with a small barrier (TS‐2) of 0.30 eV and a reaction energy of 0.04 eV. Subsequently, the vertical OH^*^ reacts with an adsorption H^*^ atom on neighbor Pt‐top site (*E*_ads_ = −0.55 eV) at the neighbor Pt‐top site to form H_2_O^*^ by overcoming a reaction barrier of 0.33 eV (TS‐3), with a reaction energy of −0.13 eV. Finally, the desorption of chemisorbed H_2_O^*^ on La‐top site has a reaction energy of 0.67 eV. Thus, the total reaction energy for the hydrogen oxidation pathway on 3O^*^‐1La/Pt(111) is −0.89 eV, with the step O^*^ + H^*^ → OH^*^ identified as the rate‐determining step (RDS), while following the Mars‐van Krevelen mechanism.^[^ ^37^ ^]^ As illustrated in Figure 3e, the catalytic oxidation of H_2_ on the Pt(111) surface tends to follow the direct oxygen dissociation pathway, with the RDS being O^*^ + H^*^ → OH^*^ as well. The corresponding reaction energy barrier is approximately ≈1.0 eV,^[^ ^51^, ^52^, ^53^ ^]^ which are much higher than that of 0.44 eV for RDS on 3O^*^‐1La/Pt(111) surface. Therefore, the active sites formed through La oxide doping significantly reduce the energy barrier of RDS and ultimately enhancing the reaction efficiency. This result demonstrates the feasibility of achieving high catalytic performance by introducing oxophilic La single atoms into the Pt active sites of zeolite catalysts. The incorporation of LaO*_x_* effectively modulates the electronic and chemical properties of the Pt surface while working as an oxygen source in the reaction. Through synergistic effects, it enhances both the catalytic oxidation performance and reaction stability.

**Figure 4:**
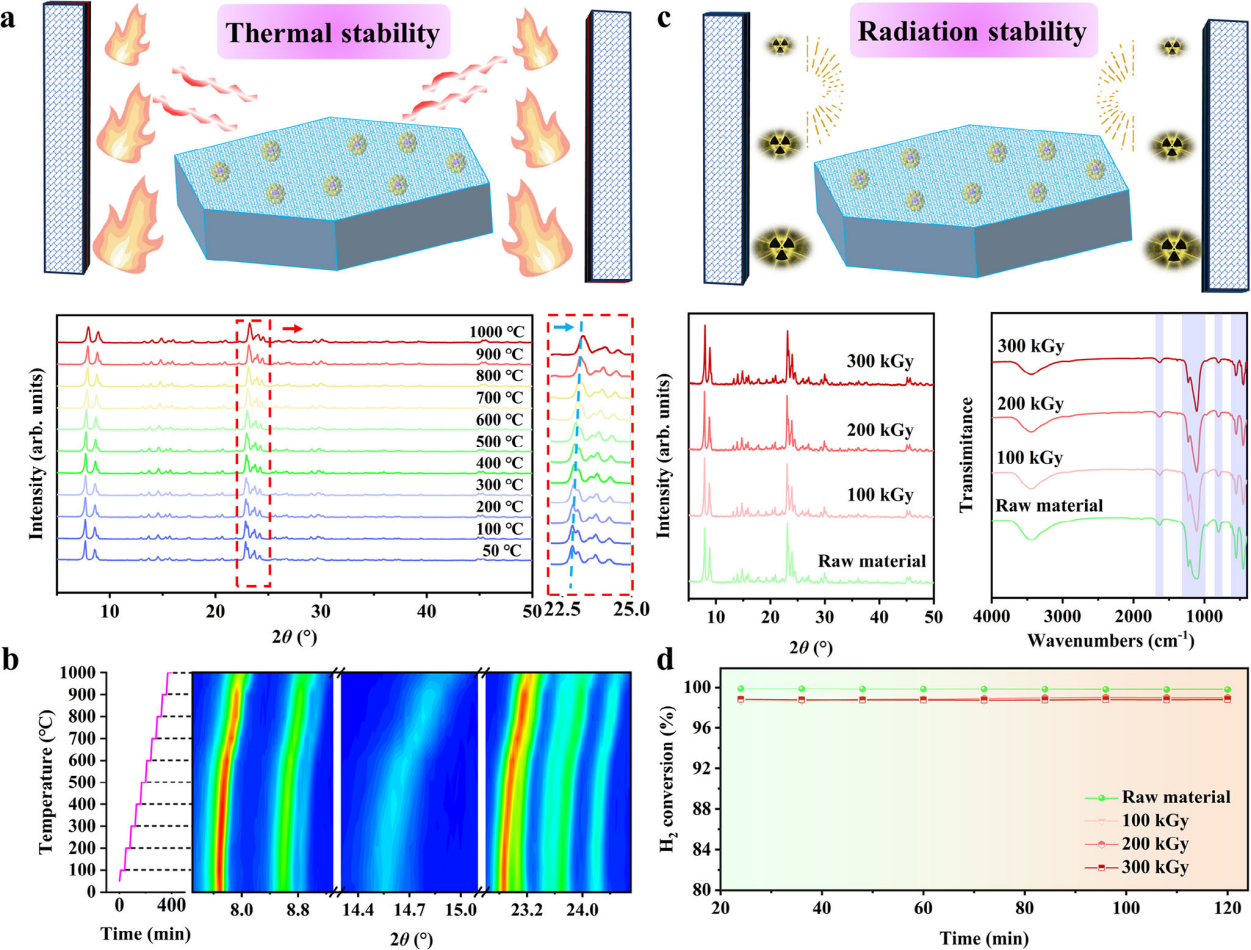
The investigations of thermal and radiation stability for PtLaO*_x_* @S‐1. a) In situ XRD patterns of the PtLaO*_x_* @S‐1 catalyst at various temperatures, b) contour plot derived from in situ XRD data, illustrating changes in peak intensity as a function of temperature for improved visualization, c) XRD patterns of PtLaO*_x_* @S‐1 before and after *β*‐irradiation at doses of 100, 200, and 300 kGy and d) a comparison of catalytic performance pre‐ and post‐irradiation. Experimental conditions: 0.05 g catalyst, 50 °C, GHSV = 48000 mL·g^−1^·h^−1^.

The thermal and irradiation stability of PtLaO*_x_* @S‐1 was also evaluated. For thermal stability, the phase evolution of PtLaO*_x_* @S‐1 was monitored at different temperatures (50–1000 °C) using in situ XRD. As shown in **Figure** **4**a, the diffraction peaks of PtLaO*_x_* @S‐1 gradually shift to higher angles with increasing the testing temperatures. This phenomenon is typically associated with a reduction in lattice parameters. According to Bragg's law, when the lattice spacing decreases, the position of the diffraction peaks shifts to higher diffraction angles.^[^ ^54^ ^]^ The lattice contraction may be induced by a slight dehydration effect under high‐temperature conditions, leading to changes in internal stress within the zeolite framework and resulting in a reduction in lattice spacing. This peak shift phenomenon is more clearly illustrated in Figure 4b. It can also be observed that when the temperature rises above 1000 °C, the intensity of the main diffraction peaks significantly decreases, indicating a decline in the crystalline quality of the zeolite catalyst. The structure stability of PtLaO*_x_* @S‐1 was further investigated in *β* irradiation field, since tritium is primarily *β‐*emitting. In previous work reported by Xu et al., 200 kGy *β* irradiation was adopted to evaluate the effect of ionizing irradiation on the hydrogen isotope catalytic oxidation capacity of Pt@CDM‐3.^[^ ^55^ ^]^ In this case, PtLaO*_x_* @S‐1 was exposed to *β*‐radiation at doses of 100, 200, and 300 kGy. The phase structure and the microscopic morphology of the catalyst showed almost no changes after irradiation (Figure 4c; Figure S22, Supporting Information). Under conditions of 50 °C and a GHSV of 48000 mL·g^−1^·h^−1^, the performance of PtLaO*_x_* @S‐1 after irradiation at 100, 200, and 300 kGy remained above 98% (Figure 4d). Compared to the performance of the non‐irradiated catalyst material (>99%), even after irradiation with 300 kGy of *β*‐radiation, the performance of PtLaO*_x_* @S‐1 decreased by only ≈1%, indicating its good radiation resistance.

## 3 Conclusion

In summary, we have for the first time demonstrated the effectiveness of monodispersed LaO*_x_* in enhancing the hydrogen isotope oxidation performance of platinum‐based zeolite catalysts. The localized electronic structure of Pt nanoclusters confined within silicalite‐1 zeolites is modulated through the incorporation of LaO*_x_*. Experimental and theoretical evidence strongly supports the correlation between LaO*_x_* incorporation into Pt nanoclusters and the enhancement of catalytic performance. The introduction of LaO*_x_* lowers the reaction energy barrier of rate‐determining step (O^*^ + H → OH^*^) from 1.00 to 0.44 eV by modulating Pt‐LaO*_x_* charge interactions and serving as an oxygen source. These effects reshape the reaction barrier and pathway, enhancing catalytic activity via the Mars‐van Krevelen mechanism. Ultimately, the PtLaO*_x_* @S‐1 catalyst maintained exceptionally high conversion rates, both after 300 kGy *β*‐irradiation and during a surprisingly long‐term restart performance test spanning ≈267 d. In addition, the performance of Ce‐ and Pr‐based catalysts confirms the broad applicability of lanthanide oxides in enhancing zeolite‐confined Pt nanocluster catalysis. This study introduces a design strategy for single‐atom oxides embedded in Pt nanocluster‐zeolite catalysts for the catalytic oxidation of hydrogen isotopes, offering an effective way to reduce the cost of precious metal catalysts in heterogeneous catalysis. The results also show the synergistic stability and activity of single‐atom oxides in zeolite‐confined metal clusters, highlighting their broad potential applications.

## 4 Experimental Section

### Materials

Tetrapropylammonium hydroxide solution (TPAOH, 25 wt.%, Shanghai McLean Biochemical Technology Co., Ltd), tetraethylorthosilicate (TEOS, Tianjin Kemio Chemical Reagent Co., Ltd), platinum chloride (PtCl_2_, Pt≥73%, Alfa Aesar), ethylenediamine (NH_2_CH_2_CH_2_NH_2_, 99%, Shanghai Aladdin Co., Ltd.), lanthanum (III) nitrate hexahydrate (La(NO_3_)_3_·6H_2_O, Shanghai Aladdin Co., Ltd.), acetylacetone (C_5_H_8_O_2_, Shanghai Aladdin Co., Ltd.), Cerium(III) nitrate hexahydrate (Ce(NO_3_)_3_·6H_2_O, Shanghai Aladdin Co., Ltd.), Praseodymium nitrate hexahydrate (Pr(NO_3_)_3_·6H_2_O, Shanghai Aladdin Co., Ltd.), and deionized water prepared using purchased instruments (Elix Water Purification System) were used as raw materials. The reagents mentioned above were used as supplied, without any additional purification. The [Pt(NH_2_CH_2_CH_2_NH_2_)_2_]Cl_2_ solution was prepared by dissolving 0.6 g PtCl_2_ in 2.5 mL ethylenediamine and 22.5 mL H_2_O mixture. The [La(acac)_3_](NO_3_)_3_ solution was prepared by dissolving 0.195 g La(NO_3_)_3_·6H_2_O in 0.5 mL acetylacetone (acac) and 4.5 mL H_2_O mixture.

### Synthesis of PtLaO*_x_* @S‐1

First, 7.5 g of deionized water and 6.5 g of TPAOH solution were mixed through centrifugation‐assisted stirring for 30 min. Subsequently, 0.5 mL of a [Pt(NH_2_CH_2_CH_2_NH_2_)_2_]Cl_2_ solution and 0.5 mL of a [La(acac)_3_](NO_3_)_3_ solution were added, followed by another 30 min of stirring. Finally, 4.16 g of TEOS was introduced, and the mixture was stirred for an additional 6 h. The resulting mixture was transferred to a 50 mL Teflon‐lined stainless‐steel autoclave and subjected to static hydrothermal treatment at 170 °C for 3 d. The obtained powder sample was washed several times with deionized water through centrifugation until the pH reached neutral. Finally, the sample was calcined at 550 °C for 6 h, followed by reduction at 400 °C for 2 h.

### Synthesis of PtCeO*_x_* @S‐1 and PtPrO*_x_* @S‐1

The synthesis of PtCeO*_x_* @S‐1 and PtPrO*_x_* @S‐1 followed the same procedure as PtLaO*_x_* @S‐1, with [La(acac)_3_](NO_3_)_3_ being replaced by [Ce(acac)_3_](NO_3_)_3_ and [Pr(acac)_3_](NO_3_)_3_, respectively.

### Synthesis of Pt@S‐1

The synthesis of Pt@S‐1 followed the same procedure as PtLaO*_x_* @S‐1, except that [La(acac)_3_](NO_3_)_3_ was not included.

### Synthesis of La@S‐1

The synthesis of La@S‐1 followed the same procedure as PtLaO*_x_* @S‐1, except that [Pt(NH_2_CH_2_CH_2_NH_2_)_2_]Cl_2_ was not included.

### Material Characterizations

The phase compositions of all the catalysts were determined using X‐ray diffraction (XRD, Haoyuan DX‐2700BH). Fourier transform infrared spectroscopy (FT‐IR) measurements were performed using a Thermo Fisher Nicolet Is10 spectrometer, covering the spectral range of 400–4000 cm^−1^. A Talos F200S field emission transmission electron microscope was used to acquire transmission electron microscopy (TEM) images and high‐angle annular dark‐field scanning TEM (HAADF‐STEM) images. High‐magnification atomic‐resolution HAADF‐STEM images were obtained using a JEM‐ARM200F transmission electron microscope equipped with aberration correction. X‐ray absorption fine structure (XAFS) measurements were conducted at the BL17B1 and BL13SSW beamlines of the Shanghai Synchrotron Radiation Facility (SSRF) in Shanghai, China, utilizing Si(111) crystal monochromators. The XAFS spectra were collected at room temperature, with extended X‐ray absorption fine structure (EXAFS) spectra recorded in transmission or fluorescence mode. Data fitting was carried out using the Artemis program within the IFEFFIT software package.^[^ ^56^ ^]^ X‐ray photoelectron spectroscopy (XPS) measurements were carried out on an FEI ESCALAB Xi+ spectrometer, with the C 1s peak at 284.8 eV used for binding energy calibration. The metal content was measured using inductively coupled plasma optical Emission spectrometry (ICP‐OES) on a PE Avio 200 instrument. Nitrogen adsorption and desorption experiments were performed at 77 K using an ASAP 2460 analyzer, with the samples pretreated under vacuum at 350 °C for degassing. Thermogravimetric and differential scanning calorimetry (TG‐DSC) measurements were conducted using a NETZSCH STA449 F3 in an air atmosphere, heating the samples from room temperature to 850 °C at a rate of 10 K min^−1^. *β‐*ray irradiation was carried out at the Sichuan Institute of Atomic Energy. The thermal stability of the catalyst in an air atmosphere was assessed using in situ XRD over a temperature range of 50–1000 °C.

### Hydrogen Isotope Catalytic Oxidation Measurements

Zero‐grade air (20 ± 2% O_2_ and the remainder N_2_, supplied by Chengdu Qiaoyuan Gas Co., Ltd.), along with high‐purity H_2_ (99.999%, Chengdu Longtai Industrial Gas Co., Ltd.) and D_2_ (99.999%, Chengdu Longtai Industrial Gas Co., Ltd.), were used to prepare a hydrogen‐oxygen mixed gas. The gas flow was regulated using an EL‐FLOW SELECT Gas Flow Meter. The catalytic reaction took place in a fixed‐bed reactor (1/4 in. OD stainless‐steel tube). The reactor was loaded with 0.05 g of catalyst and the temperature was controlled at 30, 40, 50, and 60 °C using an electric furnace (OTF‐1200X, Hefei Kejing Material Technology Co., Ltd.). The reaction outlet was analyzed online by a gas chromatograph (Agilent 7890B Gas Chromatograph). The hydrogen isotope concentration in the feed gas was maintained at 2000 ppm. The total flow rate was adjusted to 20, 30, 40, 50, and 60 mL min^−1^, corresponding to gas hourly space velocities (GHSV) of 24000, 36000, 48000, 60000, and 72000 mL·g^−1^·h^−1^. The conversion *f* (%) of H_2_ or D_2_ is defined as:

(1) $$f=\frac{C_{in}-C_{out}}{C_{in}}\times 100$$

where *C*_in_ and *C*_out_ represent the concentrations of H_2_ or D_2_ at the inlet and outlet of the reactor (in ppm), respectively.

Reaction conditions of restart performance test: 0.05 g catalyst, 50 °C, 2000 ppm H_2_ in zero air, GHSV = 48000 mL·g^−1^·h^−1^. The entire restart performance period was 384480 min (≈6408 h = ≈267 d). Inert gas was used in the dehydration process and calcination was performed at 400  C for 2 h.

### Computational Details

In this All periodic DFT calculations were carried out by using the Vienna Ab Initio Simulation Package (VASP),^[^ ^57^,^58^ ^]^ with gradient‐corrected Perdew‐Burke‐Ernzerhof (PBE) functional.^[^ ^59^ ^]^ The core‐valence interactions were treated by the projector augmented wave (PAW) method.^[^ ^60^ ^]^ Following the previous works,^[^ ^61^ ^]^ the Pt(111) surface was modeled by a p(3×3) supercell slab with five atomic layers and a vacuum thickness of 15 Å, where the bottom two layers were fixed at their bulk positions. For the La‐doped surface, one of the Pt surface atoms was replaced by one La atom. The cutoff energy of plane‐wave basis sets was set to 400 eV, and the Brillouin zone was integrated by a 3×3×1 k‐point mesh. The energies and forces of relaxations were converged to less than 10^−5^ eV and 0.02 eV Å^−1^, respectively. The reaction pathways were searched by the climbing image nudged elastic band (CI‐NEB) method,^[^ ^62^ ^]^ where the transition state was verified by the frequence analysis with only one imaginary mode. The convergence criterion for the total energy and atomic forces were set to 10^−5^ eV and 0.02 eV Å^−1^, respectively.

## Conflict of Interest

The authors declare no conflict of interest.

## Supporting information



Supporting Information

## Data Availability

The data that support the findings of this study are available from the corresponding author upon reasonable request.
