# Supplementary material for: Embedding Monodisperse LaO x Into Pt Nanoclusters for Ultra‐Stable and Efficient Hydrogen Isotope Oxidation
Source: Adv Sci (Weinh). 2025 May 29;12(30):e04224. doi: 10.1002/advs.202504224 (PMC12376614; doi:10.1002/advs.202504224)
Supplement: Supplementary file 1 — Supporting Information [file ADVS-12-e04224-s001.docx]

**Supporting Information**

**Embedding monodisperse LaO*_x_* into Pt nanoclusters for ultra-stable and efficient hydrogen isotope oxidation**

Guilin Wei ^a, b^, Jiangfeng Song ^a^, Yan Shi ^a^, Linsen Zhou ^a, *^, Xianglin Wang ^a^, Junhong Luo ^a^, Ning Liu ^b^, Feize Li ^b, *^, Xingwen Feng ^a, *^

*^a^ Institute of Materials,* *China Academy of Engineering Physics, Jiangyou, 621908, Sichuan, PR China*

*^b^ Key Laboratory of Radiation Physics and Technology of the Ministry of Education, Institute of Nuclear Science and Technology, Sichuan University, Chengdu, 610064, PR China*

* E-mail addresses of corresponding author: fengxingwen@caep.cn (X. Feng),

lifeize@scu.edu.cn (F. Li), zhoulinsen173@aliyun.com (L. Zhou)

**Supplementary Figures**

**Figure S1 |** Thermogravimetric analysis and differential scanning calorimetry (TG-DSC) result of Pt@S-1.

**Supplementary Notes**

The TG-DSC analysis presented in Supplementary Fig. 1 reveals that the thermal decomposition temperatures of ethylenediamine and TPAOH in air are 350 and 420 °C, respectively.

**Figure S2 |** The simulated X-ray diffraction (XRD) patterns of MFI-type zeolite and the experimental measured XRD patterns of Pt@S-1, La@S-1, and PtLaO*_x_*@S-1.

**Figure S3 |** Fourier Transform Infrared (FT-IR) spectra of Pt@S-1, La@S-1, and PtLaO*_x_*@S-1.

**Figure S4 |** The diagram of the testing device for evaluating the catalytic ability of the designed materials the on the oxidation of hydrogen isotope gases.

**Figure S5 |** **a** Transmission electron microscopy (TEM) image, **b** High-resolution transmission electron microscopy (HRTEM) image, **c** High-angle annular dark-field scanning transmission electron microscopy (HAADF-STEM) image and energy-dispersive spectroscopy (EDS) elemental mapping images, **d** cluster size distribution of Pt@S-1.

**Figure S6 |** **a** TEM image, **b** HRTEM image, **c** HAADF-STEM image and EDS elemental mapping images, **d** cluster size distribution of PtLaO*_x_*@S-1.


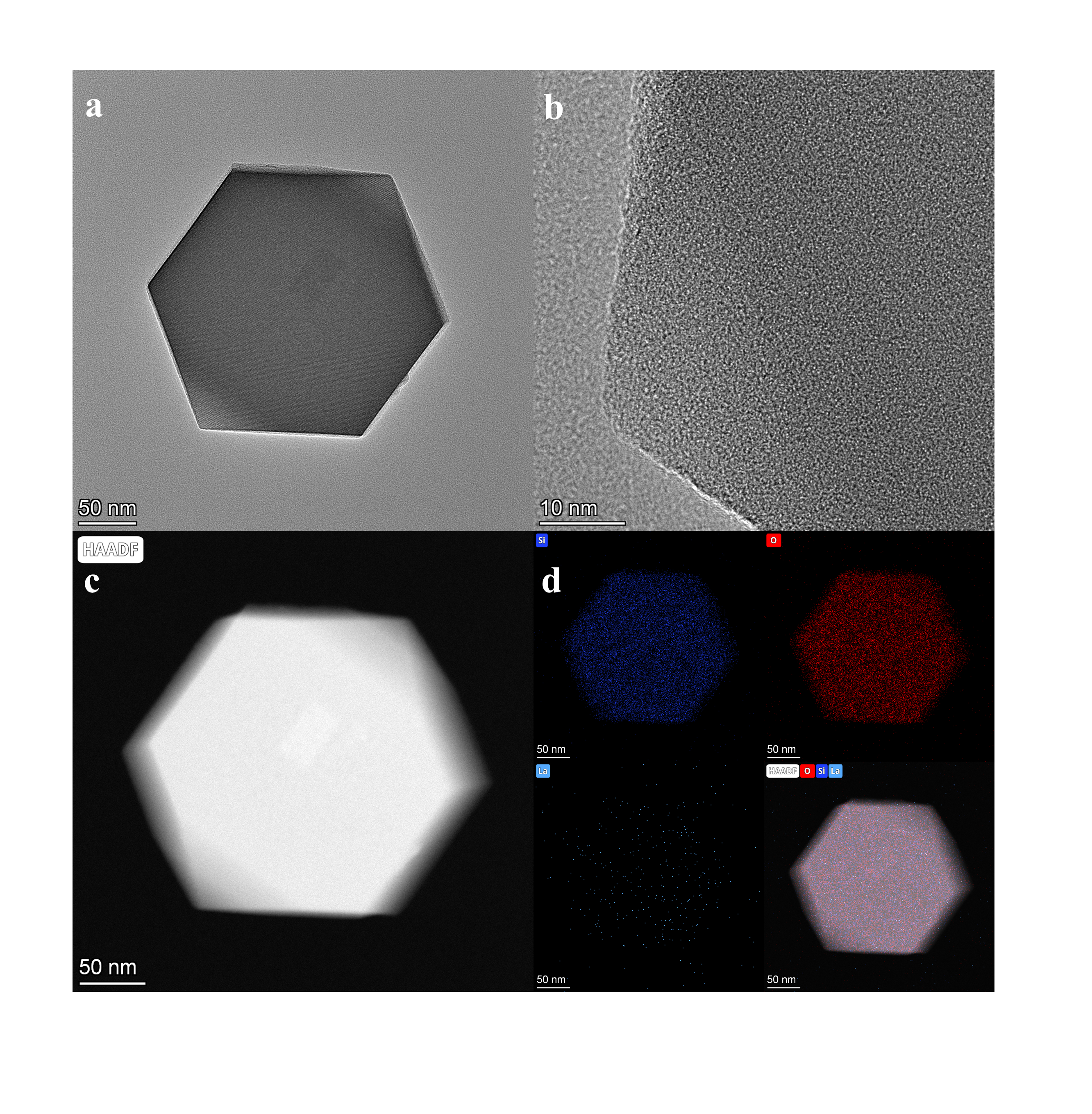


**Figure S7 |** **a** TEM image, **b** HRTEM image, **c** HAADF-STEM image and **d** EDS elemental mapping images of La@S-1.

**Figure S8 |** FT k^2^-weighted EXAFS experimental and fitting spectrum of PtLaO*_x_*@S-1 in R real space at Pt L_3_-edge.

**Figure S9 |** TEM image of PtLaO*_x_*@S-1 (inset: cluster size distribution).

**Figure S10 |** EDS elemental spectrum for PtLaO*_x_*@S-1.

**Figure S11 |** X-ray photoelectron spectroscopy (XPS) survey result for PtLaO*_x_*@S-1.

**Figure S12 |** XRD patterns of PtLaO*_x_*@S-1-used (after multiple restart performance tests).

**Figure S13 |** **a** TEM image, **b** HAADF-STEM image and EDS elemental mapping images, **c** cluster size distribution of PtLaO*_x_*@S-1-used (after multiple restart performance tests).

**Figure S14 |** XRD patterns of PtCeO*_x_*@S-1and PtPrO*_x_*@S-1.

**Figure S15 |** FT-IR results of PtCeO*_x_*@S-1and PtPrO*_x_*@S-1.

**
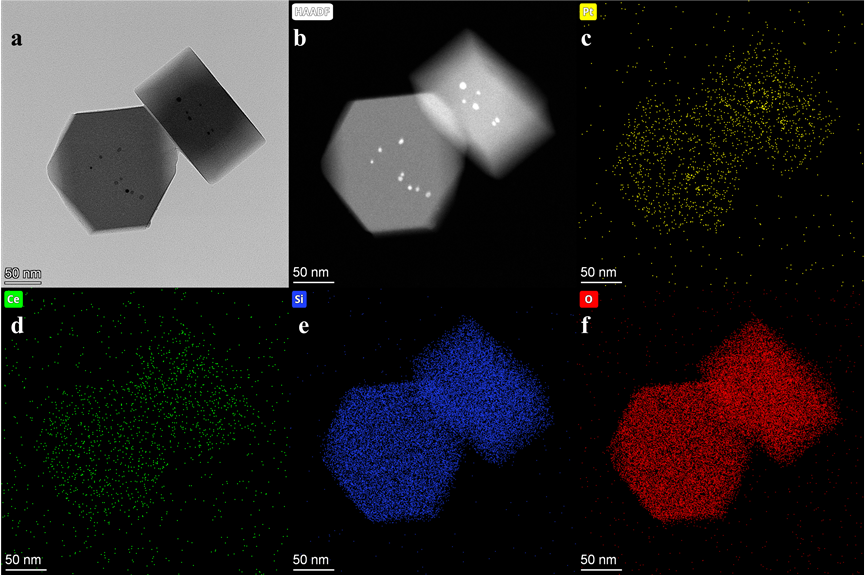
**

**Figure S16 |** **a** TEM image, **b** HAADF-STEM image and **c-f** EDS elemental mapping images of PtCeO*_x_*@S-1.

**Figure S17 |** **a** TEM image, **b** HAADF-STEM image and **c-f** EDS elemental mapping images of PtPrO*_x_*@S-1.

**Figure S18 |** Theoretical calculation model of single La doped on Pt(111) surface of PtLaO*_x_*@S-1.

**Figure S19 |** The partial density of states (PDOS) of the structure of PtLaO*_x_*@S-1.

**Figure S20 |** The charge density differences of the structure PtLaO*_x_*@S-1.

**Figure S21 |** **a** The adsorption energy of the hydroxyl molecule (OH*) on the La-top site and becomes a tilted adsorption configuration on the neighbor Pt-top site. **b** The adsorption energy of the single oxygen atom (O*) at the La-top, fcc, and hcp sites PtLaO*_x_*@S-1.


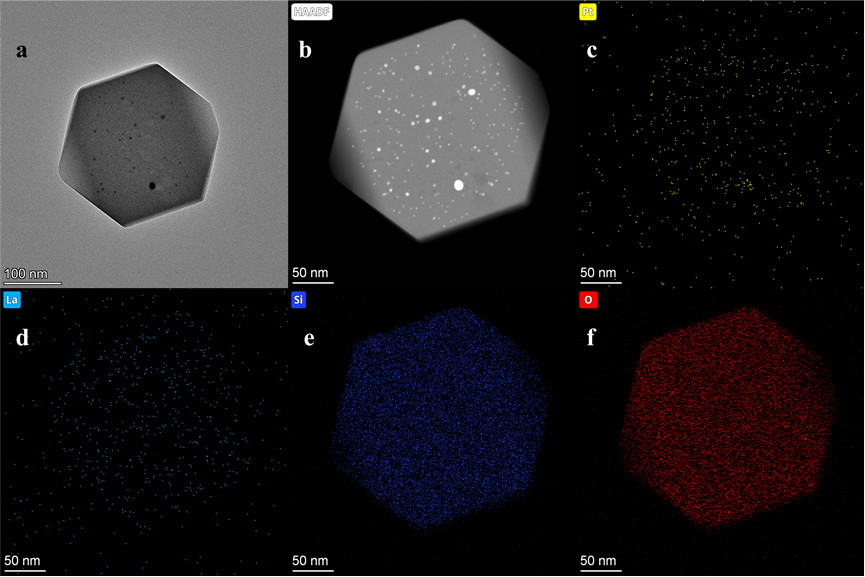


**Figure S22 |** **a** TEM image, **b** HAADF-STEM image and **c-f** EDS elemental mapping images of PtLaO*_x_*@S-1 after 300 kGy of *β*-ray irradiation.

**Supplementary Tables**

**Table S1.** Elemental analysis for different catalysts.

| Material | Pt content (wt.%) | La content (wt.%) |
| --- | --- | --- |
| Pt@S-1 | 0.56 | - |
| La@S-1 | - | 0.11 |
| PtLaO*_x_*@S-1 | 0.55 | 0.13 |

Note: The element contents of Pt and La in this table were determined by inductively coupled plasma‒optical emission spectrometry (ICP‒OES).

**Table S2.** EXAFS parameters of references and PtLaO*_x_*@S-1.

| Sample | Shell | *N*^a^ | *R* (Å)^b^ | $\sigma^{2} (Å^{2}$)^c^ | $\Delta E (eV$)^d^ | R factor^e^ |
| --- | --- | --- | --- | --- | --- | --- |
| Pt foil | Pt-Pt | 12 | 2.77 ± 0.02 | 0.004 ± 0.001 | 7.5 ± 0.3 | 0.003 |
| PtLaO*_x_*@S-1 | Pt-O  Pt-Pt | 0.6 ± 0.1 | 1.98 ± 0.03 | 0.008 ± 0.002 | 8.6 ± 0.3 | 0.003 |
|  |  | 4.7 ± 0.2 | 2.75 ± 0.02 | 0.005 ± 0.001 |  |  |

^a^ *N*, coordination number; ^b^ *R*, distance between absorber and backscatter atoms; ^c^ $\sigma^{2}$, Debye-Waller factor to account for both thermal and structural disorders; ^d^ Δ*E*, inner potential correction; ^e^ R-factor (%) indicate the goodness of the fit. The obtained *S*_0_^2^ of and Pt foil were 0.85, and it was fixed in the subsequent fitting of Pt L_3_-edge for the catalysts, respectively.

**Table S3.** A comparison of the temperature and gas hourly space velocity (GHSV) parameters of the reported catalysts and the PtLaO*_x_*@S-1 in achieving a H_2_ conversion rate of ≥99%.

| Sample | Temperature (°C) | GHSV (mL·g^−1^·h^−1^) | Ref. |
| --- | --- | --- | --- |
| Hisocat-4 | 25 | 18,000 | [1] |
| Hisocat-3 | 35 | 18,000 |  |
| Hisocat-2 | 45 | 18,000 |  |
| Hisocat-1 | 55 | 18,000 |  |
| HisoNcat-3 | 35 | 18,000 | [2] |
| Pt@CDM-1 | 45 | 18,000 | [3] |
| Pt@CDM-2 | 45 | 18,000 |  |
| Pt@CDM-3 | 45 | 18,000 |  |
| Pt/SiC washcoat | 50 | 45,000 | [4] |
| Pt@S-1/h600 | 75 | 24,000 | [5] |
| Pt/Co_3_P_4_-ZrO_2_ | 100 | 47,750 | [6] |
| Pd/Co_3_P_4_-ZrO_2_ | 100 | 47,750 |  |
| Co_3_O_4_-ZrO_2_ | 300 | 23,875 |  |
| Pd@S-1/h600 | 100 | 24,000 | [7] |
| Sn_0.95_Cu_0.05_O_2−δ_ | 320 | 30,000 | [8] |
| Ce_0.95_Cu_0.05_O_2−δ_ | 420 | 30,000 |  |
| Al_1.95_Cu_0.05_O_3−δ_ | 460 | 30,000 |  |
| Zr_0.95_Cu_0.05_O_2−δ_ | 480 | 30,000 |  |
| PtLaO*_x_*@S-1 | 50 | 72,000 | This work |

**Supplementary References**

[1] M. Xu, S. Zhang, T. Wang, B. Yu, Z. Yang, X. Wang, R. Zhou, D. Hua, *ACS Appl. Mater. Interfaces* **2022**, *14*, 7826-7835.

[2] M. Xu, F. Chen, T. Wang, B. Yu, Z. Zhao, L. Zhou, D. Hua, *ACS Appl. Nano Mater.* **2023**, *6*, 867-874.

[3] M. Xu, F. Chen, L. Zhou, Z. Xu, Q. He, D. Hua, *ACS Sustainable Chem. Eng.* **2022**, *10*, 7180-7187.

[4] A. Fernández, G. M. Arzac, U. F. Vogt, F. Hosoglu, A. Borgschulte, M. J. De Haro, O. Montes, A. Züttel, *Appl. Catal. B-Environ.* **2016**, *180*, 336-343.

[5] G. Wei, L. Zhou, J. Luo, B. Yu, F. Ding, J. Song, Y. Shi, J. Zhang, X. Feng, N. Liu, *Inorg. Chem.* **2024**, *63*, 14171-14182.

[6] S. A. Singh, K. Vishwanath, G. Madras, *ACS Appl. Mater. Interfaces* **2017**, *9*, 19380-19388.

[7] G. Wei, J. Luo, W. Yong, J. Song, Y. Shi, X. Feng, N. Liu, *J. Mater. Sci. Technol.* **2024**, *193*, 51-60.

[8] V. M. Shinde, G. Madras, *Catal. Today* **2012**, *198*, 270-279.
